# The role of cognition, affect, and resources in the influence of unreasonable tasks on work engagement: A moderated chain mediation model

**DOI:** 10.3389/fpsyg.2022.1013773

**Published:** 2022-10-06

**Authors:** Hao Cheng, Zhen Li, Junshu Zhao, Weiqiang Wang, Ruixi Zou

**Affiliations:** ^1^School of Humanities, Sichuan Agricultural University, Ya’an, China; ^2^Department of Preventive Health Care, The Fourth People’s Hospital of Ya’an, Ya’an, China; ^3^School of Business, Zhengzhou University, Zhengzhou, China

**Keywords:** unreasonable tasks, work engagement, work alienation, negative affect, supervisor support, illegitimate tasks

## Abstract

Some studies have concentrated on the adverse effects of unreasonable tasks on work engagement. So far, however, the underlying mechanisms and boundary conditions of the relationship have not been adequately discussed. Based on the cognitive-affective systems theory and the job demands-resources model, this study constructs a chain mediation model in which unreasonable tasks influence work engagement through work alienation and negative affect and explores the moderating role of supervisor support in the model. An analysis of 427 questionnaires from multiple types of organizations shows that: Unreasonable tasks have a negative impact on work engagement; work alienation and negative affect play both separate and chain mediating roles in the negative effect of unreasonable tasks on work engagement, and supervisor support negatively moderates chain mediation by moderating the positive effect of unreasonable tasks on work alienation. This study re-investigates the relationship between unreasonable tasks and work engagement from cognitive, affective, and resource perspectives, which could be a valuable addition to established research and provide suggestions and assistance for management practice.

## Introduction

In the era of fast-paced competition, it has become a way for organizations to maximize the use of human capital by having employees complete their work tasks more quickly and efficiently. Organizations or supervisors assign various work tasks to employees in order to keep the organization on track and enhance its effectiveness. However, employees actually have to deal with many unreasonable tasks in their daily work. A study found that more than half of the organizational members considered more than 11% of their daily tasks as unreasonable tasks, and even 7% of the employees considered at least 31% of their daily tasks as those (Thun et al., 2018), which indicates the prevalence of those in the workplace. The negativity of unreasonable tasks as a work stressor (Kilponen et al., 2021) has gradually attracted the attention of scholars.

The concept of unreasonable tasks comes from illegitimate tasks, which are tasks that do not meet the standards reasonably expected of employees, and unreasonable tasks, which represent a more specific meaning and refer to tasks beyond the scope of the employee’s occupation and do not meet the employee’s occupational status (Semmer et al., 2010; Semmer et al., 2019). The existing literature has clarified that factors such as abusive supervision by supervisors, inherent organizational deficiencies, leader-member exchange relationships, and leaders’ explanation of tasks can influence employees’ perceptions of unreasonable tasks (Björk et al., 2013; Sias and Duncan, 2019; Stein et al., 2020). Researchers are equally interested in the results of the effects of unreasonable tasks. Some of the studies have emphasized the adverse effects of unreasonable tasks on the individual’s intrinsic state, which include emotional exhaustion, negative affect, job satisfaction, intrinsic motivation, mental health, intention to leave academia, and meaning of work (Koch and Adler, 2018; Sonnentag and Lischetzke, 2018; Muntz et al., 2019; Pindek et al., 2019; Fila and Eatough, 2020; Muntz and Dormann, 2020; Bramlage et al., 2021; Mäkikangas et al., 2021). Another part of the research confirms that unreasonable tasks impact individuals’ external performance, such as counterproductive work behavior, sickness presenteeism, and occupational injuries (Elfering et al., 2018; Thun et al., 2018; Schulte-Braucks et al., 2019).

The presentation and validation of the above factors and results have enriched the research system on unreasonable tasks. However, this system has no more important topic than work engagement, and this is because one of the critical factors in measuring organizational productivity is the level of employee work engagement (Mihalits et al., 2021). Work engagement is a positive state that involves vigor, dedication, and absorption (Kunzelmann and Rigotti, 2021). Work engagement is also a behavioral effort that can be understood as job performance. Engaged employees are cognitively and affectively associated with their work (Bakker and Isabel Sanz-Vergel, 2013). Related studies have shown that work engagement is influenced by factors such as job demands (Schaufeli et al., 2019), job stress (Ikegami et al., 2022), and perceived insider status (Guo et al., 2022). Specific to unreasonable tasks, they are a hindrance stressor and offend the employee’s professional role, making it difficult for employees to be engaged in their work. Several studies found that unreasonable tasks have a negative impact on work engagement, based on the perspectives of the self-determination theory, the job demands-resources model, and the theory of Stress as Offence to Self (van Schie et al., 2014; Schmitt et al., 2015; Kilponen et al., 2021). Admittedly, these studies have elevated the status of work engagement in the field related to unreasonable tasks. However, these studies are not sufficiently persuasive. This can be explained by the fact that the process by which unreasonable tasks influence work engagement is not clearly presented in these studies. Scholars have not explored the mechanisms by which unreasonable tasks influence work engagement, nor have they clarified the boundary conditions of the relationship, and they lack a deeper understanding of the relationship. In fact, the change in behavior is not the most direct response when employees face work stress, like unreasonable tasks. Studies on the direct effect of unreasonable tasks on work engagement are insufficient to explain the relationship between the two, and there may be a “black box” between the two. Therefore, the purpose of this study is to better understand the mechanism by which unreasonable assignments affect work engagement, as well as its boundary conditions.

The cognitive-affective systems theory suggests that an individual’s internal cognitive or affective system will be activated in a particular external situation, in turn leading to the individual’s behavior (Mischel and Shoda, 1995).

The study assumes that work alienation as a cognitive variable is vital in the process of unreasonable tasks affecting work engagement. Work alienation reflects the separation of employees from their work at the cognitive level (Nair and Vohra, 2010). Employees cannot develop work alienation without factors related to work stress (Durrah, 2020). Meanwhile, established studies have shown that work alienation contributes to some negative outcomes such as emotional exhaustion (Yu et al., 2021), knowledge hiding (Guo et al., 2021), and inhibits positive aspects such as voice behavior (Deniz and Cimen, 2022), job performance (Amarat et al., 2019). According to the cognitive-affective systems theory, unreasonable tasks that exceed the employee’s occupational scope and do not fit with the employee’s occupational expectations can trigger alienation from the work and the environment at the cognitive level, which in turn affects work engagement. Therefore, work alienation can reflect the processing of employees’ cognitive systems in the process of unreasonable tasks affecting work engagement.

In addition, this study considers negative affect as a mechanism by which unreasonable tasks affect work engagement, and this is because employees’ affect is an important outcome when faced with work stress and negative work events. Negative affect is a transient state of affect triggered by negative work events (Fisher, 2000). It is an affective reaction that occurs when employees are faced with unreasonable tasks (Fila and Eatough, 2020), stress (Horiuchi et al., 2018), and work interruption (Sonnentag et al., 2018). Negative affect also adversely influences work engagement (Fan, 2022), work effort (Anjum et al., 2022), and job satisfaction (Lan et al., 2022). Based on the cognitive-affective systems theory, negative affect triggered by unreasonable tasks can cause employees to reduce positive work behaviors such as work engagement. Therefore, the effect of unreasonable tasks on work engagement can be explained by negative affect.

Another perspective of the cognitive-affective systems theory is that external situations can trigger the processing of both cognitive and affective systems, i.e., situations activate the cognitive system, which in turn evokes the affective system and ultimately influences the occurrence of individual behaviors and attitudes (Mischel and Shoda, 1995). The specific application of this perspective in this study is that unreasonable tasks trigger work alienation among employees, which in turn generates negative affect and ultimately reduce their work engagement.

Therefore, based on the cognitive-affective systems theory, this study takes a composite perspective of cognition and affect, sets the focus on two variables, work alienation and negative affect, and constructs a chain mediation model to answer the first research question: What are the intrinsic mechanisms between unreasonable tasks and work engagement?

In addition, according to the job demands-resources model, resources are able to successfully reduce the adverse impacts of job demands (Xanthopoulou et al., 2007). Job resources can weaken the adverse effects and help employees when faced with unreasonable tasks (Mäkikangas et al., 2021). This study proposes that supervisor support as a boundary condition can moderate the negative effects of unreasonable tasks. Supervisors implement organizational decisions and hold many work resources (Chae et al., 2019). Employees receive work resources when they receive supervisor support (Huo and Jiang, 2021). Supervisor support is the employee’s perception of the supervisor’s support for all aspects of the employee, recognition of contributions, and concern for wellbeing (Eisenberger et al., 2002). Supervisor support provides resources to employees through instrumental help and affective support (Zhu et al., 2019). Instrumental help can bring material, interpersonal, and work resources to compensate for employees’ resource depletion, help employees do their jobs better, and reduce various negative effects caused by unreasonable tasks as job demands. Affective support is beneficial in increasing employees’ psychological resources, enhancing their job satisfaction (Sargent and Terry, 2000), and making employees feel internally safe, which makes them more engaged in their work (Kolodinsky et al., 2018). This suggests that supervisor support may effectively reduce employee work alienation and negative affect and promote work engagement. Therefore, this study will further explore the second research question: Does supervisor support moderate the chain mediating effect of work alienation and negative affect?

In summary, based on the cognitive-affective system theory, this study combines the job demands-resources model to construct a chain mediation model in which unreasonable tasks affect employees’ work engagement through work alienation and negative affect and to investigate the moderating role of supervisor support on the chain mediation role.

The study has several contributions. Firstly, it confirms that unreasonable tasks negatively influence work engagement in different types of organizations in China. Secondly, unlike previous studies, we reveal that work alienation and negative affect are transmission mechanisms between unreasonable tasks and work engagement, fully explaining how unreasonable tasks have a negative impact on work engagement and expanding the system of the impact outcomes of unreasonable tasks and the system of the impact factors of work engagement. Thirdly, the study found that supervisor support as a boundary condition can effectively moderate the chain mediation, which led to an in-depth understanding of why employees are engaged in their work even when faced with unreasonable tasks. Lastly, it is also significant that the study focused on the individual construct of the unreasonable tasks rather than on the illegitimate tasks as a whole, which was beneficial in discovering the specific qualities of the sub-dimensions of the illegitimate tasks.

## Research hypothesis

### Unreasonable tasks and work engagement

Unreasonable tasks are tasks that are not appropriately asked of a specific person, specifically, tasks that are outside the scope of their professional role and not in line with their professional status (Semmer et al., 2010; Semmer et al., 2015). There are clear criteria for discerning unreasonable tasks, and one of the following conditions is met: First, the task should be performed by someone else. For example, an employee of human resource management is asked to do temporary reception work at the front desk; second, the task is beyond the defined scope of responsibility or does not match the employee’s experience. For example, the organization lets the grassroots employees handle the work that only the managers have the authority to do, or the organization gives the company’s annual training program to the new employees who have just graduated from college to complete; third, the task will put the employees in an embarrassing situation. For example, the leader arranges for subordinates to inform and criticize employees who do not meet performance standards in a general meeting; fourth, the task is perceived as unfair. For example, the organization arranges for other employees to perform simple tasks while they are assigned very demanding tasks.

Work engagement is a work-related, positive, and satisfying psychological state that does not focus on any particular object, event, individual, or behavior. It encompasses three dimensions: work vigor, work dedication, and work absorption. Work vigor means that employees are energetic at work, psychologically resilient, willing to put effort into their work, and able to persevere even in the face of difficulties. Work dedication means that individuals feel meaningful, enthusiastic, inspired, proud, and challenged by their work. The last dimension of work engagement is work absorption, which refers to employees’ complete immersion in their work, employees’ difficulty in taking time off from work to do other things, and employees’ feeling that time passes quickly (Schaufeli et al., 2002).

The definitions of unreasonable tasks and work engagement show that they have opposite attributes and may have a negative relationship with each other. According to the job demands-resources model, excessive job demands and a lack of job resources can lead to adverse effects, and high levels of combined job demands are negatively associated with work engagement (Riedl and Thomas, 2019). Unreasonable tasks are unconventional job demands made by the organization or leader on employees. Employees may need more job and psychological resources to deal with unreasonable tasks. However, due to the limited nature of personal resources, employees may preserve the resources by retaining energy, and thus employees reduce work engagement.

Furthermore, it has been argued that hindrance stress can make employees believe that the stressful event cannot lead to any opportunity for personal improvement (Inam et al., 2021), while unreasonable tasks as hindrance stress (Schmitt et al., 2015) can also hinder personal development. Therefore, owing to the hindering nature of unreasonable tasks, employees have difficulty having positive attitudes and perceptions toward the organization or their supervisors when dealing with them. They begin to lack motivation and energy for their work, thus reducing their work engagement.

In summary, this study proposes the following hypothesis:

*H1: Unreasonable tasks negatively affect work engagement.*

### Mediating role of work alienation

Work alienation represents an individual’s estrangement and disconnection from self, work, and the relevant environment (Usman et al., 2020). Unreasonable tasks convey information and contain characteristics that cause employees to be alienated from their work. In this state, employees cannot integrate into the work and the environment, and the state of vigor, dedication, and absorption of employees are also difficult to sustain, so it is hard for employees to engage in work.

The cognitive-affective systems theory suggests that situational factors drive the processing of the individual’s cognitive system, in turn leading to rational thinking and, ultimately, the individual’s behavior and attitudes (Mischel and Shoda, 1995).

On the one hand, some work situation characteristics can trigger employees’ work alienation, such as organizational justice, work role clarity, and job autonomy. The work environment reflected in the unreasonable tasks has such characteristics. First, as shown earlier, unreasonable tasks represent injustice in the organization, where tasks and resources are not distributed fairly, and employees are not equally rewarded. Unfair treatment can lead to the alienation of employees from their work (Durrah, 2020). A study by Sulu et al. (2010) on Turkish healthcare workers has confirmed that distributive and procedural injustice are antecedents of work alienation. Second, a study by Björk et al. (2013) states that illegitimate tasks represent deficient organizational control, which implies that unreasonable tasks also mean a lack of norms and low formality in the organization, which can cause employees to have difficulty in clarifying their roles and the importance of their roles, thus creating estrangement and disconnection from work (Usman et al., 2020). Third, it is difficult for employees to participate in the decision-making process of task allocation. Even if employees can participate in it, their opinions and suggestions are not easily adopted by the supervisor and the organization, and they can hardly influence the decision results. At the same time, when the supervisor or organization assigns unreasonable tasks, employees can only accept those due to their subordinate status and the lack of their resources. In both cases, employees lack work autonomy and are powerless to change the outcome of their task assignment, thus becoming alienated from their work (Ali et al., 2022).

On the other hand, unreasonable tasks do not reduce employees’ core tasks but rather take away part of the resources for employees to complete their core tasks and hinder the completion of their work (Schulte-Braucks et al., 2019), and in turn, employees may perceive unreasonable tasks as meaningless for their career development. In particular, employees may perceive unreasonable tasks that do not match their experience and abilities as unchallenging and meaningless for their professional growth. This sense of meaninglessness can alienate employees from work (Nair and Vohra, 2010; Peng et al., 2022).

The cognitive-affective systems theory suggests that individual cognitive systems activated by external situational factors drive corresponding behaviors and attitudes (Mischel and Shoda, 1995). Studies conducted by Usman and other scholars in the service and manufacturing industries suggest that work alienation is positively associated with work burnout (Usman et al., 2020). Work engagement, the antithesis of work burnout (Schaufeli et al., 2019), may have a negative relationship with work alienation. Based on this background, it can be said that unreasonable tasks act as situational factors to motivate employees to develop work alienation and that this estrangement from work and the environment drives employees to behave in ways that reduce their work engagement.

Therefore, after unreasonable tasks stimulate employees to develop work alienation, employees reduce their work engagement as a subsequent manifestation. Firstly, work alienation is characterized by the psychological separation of employees from their work. In this state, the ability to obtain physical and mental energy decreases, the body becomes tired, the spirit begins to degenerate (Khan et al., 2019), and the attitude toward things around them becomes negative. As a result, employees lack the energy and vigor to get involved in their work and cannot work with a spirit of effort. Secondly, work alienation can make employees think mundanely and believe that work is only a means of obtaining money. Focusing only on the material rewards that come from work, employees can no longer feel proud and inspired by their work and find it difficult to approach work with a mindset of dedication. Finally, work alienation often means a lower motivation to work (Sulu et al., 2010). High work motivation can lead to positive work behaviors and attitudes. In contrast, low work motivation only motivates employees to adopt negative behaviors, such as avoidance at work and distraction at work.

In summary, this study proposes the following hypotheses:

*H2a: Unreasonable tasks positively affect work alienation.*

*H2b: Work alienation negatively affects work engagement.*

*H2c: Work alienation mediates the relationship between unreasonable tasks and work engagement.*

### Mediating role of negative affect

Negative affect is a typical type of affect, generally manifested as anger, anxiety, disgust, fear, and other affect (Liu et al., 2007). Unreasonable tasks, as external contextual factors, can interfere with employees’ core tasks, take up their time and resources, and convey injustice information, thus triggering negative affect such as anger and disgust, resulting in low productivity, avoidance, and thus difficulty in work engagement.

The cognitive-affective systems theory suggests that under the influence of external situations, an individual’s affective system is stimulated and processed (Mischel and Shoda, 1995). Negative affect can be reflected in the processing of affective units under the influence of external factors such as unreasonable tasks. Unreasonable tasks lead to negative affect on employees in the following ways: First, leaders assigning unreasonable tasks to employees will undoubtedly increase their workload. Suppose the task is difficult or new to the employee. In that case, it requires the employee to learn new knowledge and skills to handle it, which distracts the employee from the existing task (Elfering et al., 2018) and interrupts the completion of their work, which in turn generates negative affect (Sonnentag et al., 2018). Second, core tasks are the focus of daily work. However, unreasonable tasks, as non-core tasks, will undoubtedly consume time resources that employees would otherwise use to complete core tasks, making it difficult for employees to complete their work (Schulte-Braucks et al., 2019), forcing employees to speed up their work or sacrifice non-work time to complete core tasks (Zhou et al., 2020). Employees’ time resources are depleted, creating time pressure and resulting in negative affect (Wang et al., 2021). Finally, unreasonable tasks also represent a lack of organizational justice to some extent. When employees receive work that others should have handled, they inevitably develop a mentality of questioning the justice of organizational procedures and interactions. Negative affect is the most direct reaction of employees when they are treated unfairly by the organization (Ahmed et al., 2018). Unreasonable tasks have been demonstrated to have a significant positive relationship with negative affect (Pindek et al., 2019).

Based on the cognitive-affective systems theory, the affective system drives employees to behave and attitude accordingly when influenced by external situations (Mischel and Shoda, 1995). The perspective can be explained by its specific application to the present study: employees develop negative affect in organizations that create hindrance stress, which results in negative work attitudes and low work engagement (Inam et al., 2021). Engaged individuals are energetic and able to establish an effective connection with their work (Schaufeli et al., 2006), while negative affect, as a negative experience felt by the individual, is incompatible with the state of work engagement. On the one hand, the individual’s psychological, social, and physical resources are conducive to fostering good work behaviors and attitudes, and positive affect can play a role in maintaining these resources. On the contrary, negative affect cannot maintain individual resources, which can reduce employees’ motivation and interfere with their concentration (Li et al., 2021). On the other hand, employees experience discomfort when they have negative affect and may withdraw actively from negative situations to reduce their experience of negative affect. For example, when employees experience fearful negative affect, individuals receive signals to flee, actively avoid potential harm (Isgett and Fredrickson, 2015) and find ways to engage in avoidance behaviors (Ilies and Judge, 2005). Reducing work engagement is one of the ways that employees actively avoid negative situations (Wang and Shi, 2022) and is an affectively driven behavior in the cognitive-affective systems theory.

In summary, this study proposes the following hypotheses:

*H3a: Unreasonable tasks positively influence negative affect.*

*H3b: Negative affect negatively influences work engagement.*

*H3c: Negative affect mediates the relationship between unreasonable tasks and work engagement.*

### Chain mediating effects of work alienation and negative affect

Cognitive-affective systems theory suggests that individual behavior and attitudes are not always directly influenced by external contexts. The theory incorporates two types of individual characteristics—cognition and affect into the theoretical model and believes that the individual’s cognitive unit or affective unit is driven by external situations and thus influences individual behavior and attitudes. However, an individual’s cognitive and affective units are not independent, and they can interact with each other. External situational factors first activate the cognitive system, which in turn evokes the affective system and ultimately leads to individual behaviors and attitudes (Mischel and Shoda, 1995). Work alienation reflects the employee’s cognitive psychological state of separation from work, which is a cognitive factor. At the same time, negative affect belongs to affective factors and reflects how individuals feel when they are in a difficult situation.

As an external situational factor, unreasonable tasks can induce employees’ work alienation, in turn resulting in negative affect, and eventually, employees will take action to reduce work engagement. Based on the cognitive-affective system mechanism, unreasonable tasks arranged by the organization or supervisor can cause employees to evaluate such tasks, the organization, and the supervisor negatively, such as questioning the meaning of the task, the fairness of the organization, and the support of the supervisor, and then employees begin to have cognitive-level separation from the work as a whole. When employees begin to alienate from their work, they not only persistently evaluate it negatively, but their affect change (Khan et al., 2019). Employees develop negative affect such as anger and disgust, thus antagonizing the work situation on an affective level. Negative affect dampens employees’ motivation and leads to thoughts of avoiding work situations, which ultimately makes it difficult for employees to engage in their work.

In summary, this study proposes the following hypothesis:

*H4: There is a chain mediating effect of work alienation and negative affect between unreasonable tasks and work engagement.*

### Moderating role of supervisor support

Supervisor support has been explained as an employee’s perception that supervisor values his or her work contribution and cares about his or her wellbeing (Kottke and Sharafinski, 1988), specifically, the material, interpersonal, and work-related supportive resources that employees perceive from their supervisors. These include higher compensation packages, good communication, autonomous authority and experiential guidance in handling work tasks, concern for subordinates’ physical and mental health, and other support. According to previous studies, supervisor support can provide more resources to help employees perform their tasks, which is the opposite of the negative messages sent by unreasonable tasks, thus buffering the damage caused by unreasonable tasks (Fila and Eatough, 2020).

One hypothesis of the job demands-resources model treats job resources as a boundary condition and considers job resources to mitigate a range of adverse effects of high job demands on employees (Xanthopoulou et al., 2007). Unreasonable tasks require more psychological and work resources as a special job demand. The higher the degree of supervisor support, the more resources employees receive, and the more they can reduce and compensate for the resource depletion caused by unreasonable tasks, thus reducing the negative effects. Therefore, this study concluded that supervisor support as a boundary condition could weaken the negative effects of unreasonable tasks.

Lack of supervisor support can reinforce employees’ alienation from their work and environment (Usman et al., 2020), and adequate supervisor support can help employees integrate into their work. A higher level of supervisor support means good communication between supervisors and employees, which can steer employees’ perceptions of unreasonable tasks toward rationality. Through sincere communication with employees, supervisors understand employees’ needs, analyze why employees feel alienated from work, and explain to employees the distinctiveness and importance of unreasonable tasks to the organization. Therefore, employees’ understanding of the organization will be strengthened, and the loss of psychological resources caused by unreasonable tasks will be reduced so that employees will perceive the meaning of unreasonable tasks and ultimately reduce work alienation. In addition to good communication, it is also profitable for supervisors to provide employees with work resources to deal with unreasonable tasks. Research has shown that job autonomy as a job resource influences work alienation (Vanderstukken and Caniels, 2021). The autonomous power granted by the supervisor to the employee can stimulate the employee’s independent will, reinforce the employee’s sense of self-control over the work, and then restrain the employee’s alienation from the work. In addition, when supervisors provide employees with guidance and assistance in handling unreasonable tasks, the depletion of employees’ work resources will be diminished accordingly, and employees will complete unreasonable tasks faster and with higher quality. As a result, employees can devote more work resources to their core tasks, thus enhancing the meaning of work and minimizing work alienation.

In addition, this study applies the job demands-resources model to the hypothesis of moderated chain mediation. This study suggests that resources from supervisor support can not only reduce work alienation but also have an impact on negative affect and work engagement. Supervisor support as a resource can stimulate employees’ rational perception of work tasks, which is conducive to triggering individual affective system processing and, ultimately, behavior and attitude change. Specifically, supervisors give employees supportive resources to deal with unreasonable tasks, which can effectively compensate for employees’ resource depletion and enable them to view such work events rationally and treat their work with a normal mind. Thus, employees will not get stuck in the mire of negative affect from which they cannot extricate themselves and continue to stay engaged in their work.

In summary, this study proposes the following hypotheses:

*H5a: Supervisor support negatively moderates the positive relationship between unreasonable tasks and work alienation.*

*H5b: Supervisor support moderates the chain mediating effect of work alienation and negative affect on the relationship between unreasonable tasks and work engagement by moderating the positive effect of unreasonable tasks on work alienation.*

## Materials and methods

### Participants and procedures

To test this study’s hypotheses and make the data more convincing, we surveyed different types of organizations in several provinces and cities in China. These organizations come from various industries, including agriculture, service, the Internet, real estate, manufacturing, and volunteerism. The surveyed organizations include state-owned enterprises, private enterprises, foreign-invested enterprises, Sino-foreign joint ventures, and government departments. This study used online and offline methods to collect primary data. The research team made several trips to two private agricultural and livestock enterprises in Sichuan Province to conduct investigations. Due to COVID-19, it was difficult for the research team to travel to other regions to conduct the research, so we contacted several organizations online in Yunnan, Shenzhen, Chongqing, and other provinces and cities. The managers of the organizations that agreed to cooperate with the research helped us contact the respondents. They provided them with a survey instruction on our behalf, including the implementation organization, purpose, process, and information protection commitment of this research.

The whole survey started in November 2021 and ended in March 2022. A two-round survey was conducted for this study to reduce common method variance. In the first round, we surveyed 506 respondents, and we asked respondents to complete demographic information, an unreasonable task scale, and a supervisor support scale, resulting in 464 respondents providing complete data. Half a month later, we conducted a second round of the survey. With 464 respondents, we asked the respondents to fill in the information on three variables: negative affect, work alienation, and work engagement. The research team followed the screening criteria, such as inconsistency and omission, to exclude the questionnaires that did not meet the requirements and ultimately obtained 427 valid questionnaires. The sample characteristics are shown in Table 1.

**TABLE 1:** Sample characteristics.

Characteristics	Options	Frequency	Percentage (%)
**Sex**	Male	232	54.3
	Female	195	45.7
**Age**	16–19	11	2.6
	20–29	112	26.2
	30–39	128	30.0
	40–49	112	26.2
	50 and above	64	15.0
**Education level**	Below college degree	26	6.1
	college degree	216	50.6
	Bachelor’s degree	140	32.8
	Master’s degree and above	45	10.5
**Job tenure**	Within 1 year	25	5.9
	1–5 years	102	23.9
	6–10 years	109	25.5
	11–15 years	117	27.4
	16–20 years	38	8.9
	More than 20 years	36	8.4
**Organization type**	Government departments	52	12.2
	State-owned enterprises	100	23.4
	Private enterprises	109	25.5
	Foreign-invested enterprises	86	20.1
	Sino-foreign joint ventures	70	16.4
	Others	10	2.3

### Variable measurement

This study used mature scales to ensure the validity of the measurement tool. Furthermore, the research team used various techniques, such as translation and back-translation, to make sure that the Cross-Cultural Scale was valid. The study used a 5-point Liker scale ranging from 1 (strongly disagree) to 5 (strongly agree). With reference to previous studies (Schmitt et al., 2015), the control variables were set as sex, age, education level, and job tenure.

Unreasonable tasks were measured by the Bern Illegitimate Task Scale (Semmer et al., 2010), which has eight questions, of which the unreasonable task dimension accounts for four questions, such as “Do you have work tasks to take care of, which you believe should be done by someone else?” The Cronbach’s α was 0.872.

Negative affect was measured by a scale developed by Liu and other scholars (Liu et al., 2007). The scale has good reliability in both Chinese and American tests. The scale has five questions, such as “My job makes me angry.” The Cronbach’s α was 0.883.

Work alienation was measured by a scale developed by Nair and Vohra with 8-grid questions (Nair and Vohra, 2010), such as “Over the years, I have become disillusioned about my work.” The Cronbach’s α was 0.898.

Work engagement was measured by a short version of a nine-item scale developed by Schaufeli and other scholars (Schaufeli et al., 2006) with questions such as “I am immersed in my work.” The Cronbach’s α was 0.927.

Supervisor support was measured using a scale developed by Cheng et al. (2003) with four questions, such as “My supervisor offers help when I am in a personal crisis.” The Cronbach’s α was 0.895.

## Data analysis and results

### Common method variance testing

In this study, the CMV-ULMC method was used to test the common method variance through AMOS 26. We found that the model controlling the common method factors did not significantly improve the fitting effect (Δχ^2^/df = 0.019, ΔCFI = 0.003, ΔTLI = 0.001, ΔIFI = 0.003, ΔRMSEA = 0). Therefore, there is no serious common method variance.

### Correlation analysis

This study used SPSS 28 for correlation analysis. The M, SD, and correlation coefficients are shown in Table 2. The results showed that unreasonable tasks were significantly negatively correlated with work engagement (*r* = −0.503, *p* < 0.01), unreasonable tasks were significantly positively correlated with work alienation and negative affect (*r* = 0.502, *p* < 0.01; *r* = 0.471, *p* < 0.01), work alienation and negative affect were significantly negatively correlated with work engagement (*r* = −0.504, *p* < 0.01; *r* = −0.543, *p* < 0.01), and supervisor support was significantly negatively related to work alienation and negative affect (*r* = −0.272, *p* < 0.01; *r* = −0.208, *p* < 0.01). The above results provided support for the subsequent hypothesis testing.

**TABLE 2:** Correlation analysis.

Variables	Sex	Age	Edu	JT	URT	WA	NA	SS	WE
**Sex**	1								
**Age**	0.007	1							
**Edu**	–0.038	0.004	1						
**JT**	0.075	0.183**	–0.028	1					
**URT**	–0.034	−0.185**	0.049	–0.057	1				
**WA**	0.005	–0.080	0.095*	–0.034	0.502**	1			
**NA**	–0.028	−0.161**	0.112*	–0.032	0.471**	0.515**	1		
**SS**	0.083	–0.005	–0.009	–0.001	−0.174**	−0.272**	−0.208**	1	
**WE**	0.045	0.147**	−0.135**	0.020	−0.503**	−0.504**	−0.543**	0.250**	1
**M**	1.457	3.248	2.478	3.349	2.291	2.396	2.373	3.597	3.306
**SD**	0.499	1.081	0.764	1.321	1.083	0.981	1.081	1.058	1.215

*N* = 427; EDU indicates education level; JT indicates job tenure; URT indicates unreasonable tasks; WA indicates work alienation; NA indicates negative affect; WE indicates work engagement; SS indicates supervisor support, same below. *p < 0.05; **p < 0.01, same below.

### Measurement model

The convergent validity has been extensively validated due to the relatively mature nature of the scale used. In this study, AMOS 26 was used to test the discriminant validity. As shown in Table 3, the five-factor model fitted best (Δχ^2^/df = 2.010, CFI = 0.950, TLI = 0.945, IFI = 0.950, RMSEA = 0.049), indicating good discriminant validity among the variables.

**TABLE 3:** Confirmatory factor analysis (CFA) results.

Models	χ^2^	df	χ^2^/df	CFI	TLI	IFI	RMSEA
**Five-Factor**	793.830	395	2.010	0.950	0.945	0.950	0.049
**Four-Factor**	1429.447	399	3.583	0.871	0.859	0.871	0.078
**Three-Factor**	1935.572	402	4.815	0.807	0.792	0.808	0.095
**Two-Factor**	2890.210	404	7.154	0.688	0.664	0.629	0.120
**One-Factor**	3888.163	405	9.600	0.562	0.530	0.564	0.142

*N* = 427; One-factor model (URT + WA + NA + WE + SS); two-factor model (URT + WA + NA + WE, SS); three-factor model (URT + WA + NA, WE, SS); four-factor model (URT, WA + NA, WE, SS); five-factor model (URT, WA, NA, WE, SS).

### Hypothesis testing

**FIGURE 1:**
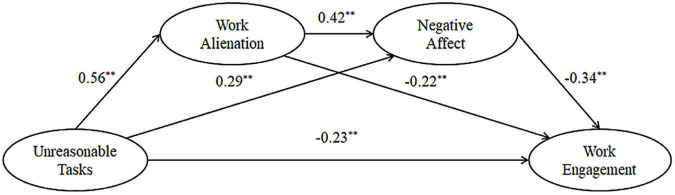
Path analysis diagram (*N* = 427).

In this study, competitive models were constructed using AMOS 26 to compare with the hypothetical model so that a path analysis could be performed to test the relationship between the variables. As shown in Table 4, the best fit between the observed data and the hypothetical model was found. The results of the path analysis with the hypothetical model are shown in Figure 1.

(1) The standardized path coefficient of unreasonable tasks affecting work engagement was −0.23 (*p* < 0.01), showing that unreasonable tasks had a significant negative predictive influence on work engagement.
(2) The standardized path coefficient of unreasonable tasks affecting work alienation was 0.56 (*p* < 0.01), showing that unreasonable tasks had a significant positive predictive influence on work alienation.
(3) The standardized path coefficient of work alienation affecting work engagement was −0.22 (*p* < 0.01), showing that work alienation had a significant negative predictive influence on work engagement.
(4) The standardized path coefficient of unreasonable tasks affecting negative affect was 0.29 (*p* < 0.01), showing that unreasonable tasks had a significant positive predictive influence on negative affect.
(5) The standardized path coefficient of negative affect on work engagement was −0.34 (*p* < 0.01), showing that negative affect has a significant negative predictive influence on work engagement.
(6) The standardized path coefficient of work alienation affecting negative affect was 0.42 (*p* < 0.01), showing that work alienation has a significant positive predictive influence on negative affect.

**TABLE 4:** Model fitness index.

Models	χ^2^	df	χ^2^/df	CFI	TLI	IFI	RMSEA
**Hypothetical model**	631.921	293	2.157	0.950	0.945	0.950	0.052
**Competitive model 1**	711.904	296	2.405	0.939	0.933	0.839	0.057
**Competitive model 2**	670.674	295	2.273	0.945	0.939	0.945	0.055
**Competitive model 3**	657.858	294	2.238	0.946	0.941	0.947	0.054
**Competitive model 4**	646.835	294	2.200	0.948	0.942	0.948	0.053

Therefore, H1, H2a, H2b, H3a, and H3b were supported.

Bootstrap repeated sampling was performed using the PROCESS macro, and 95% confidence interval (CI) and 5,000 repetitions of sampling were performed to test the mediating effect of work alienation and negative affect on the relationship between unreasonable tasks and work engagement. Results are shown in Table 5. The indirect effect value of work alienation between unreasonable tasks on work engagement was −0.107, and the 95% CI [−0.166 to −0.052] did not contain 0, indicating a significant mediating effect of work alienation between unreasonable tasks and work engagement. Thus, H2c was supported.

**TABLE 5:** Results of bootstrap test for mediating effects.

Effect types	Effect value	Boot SE	Bootstrap 95% CI	
			LLCI	ULCI
**Total indirect effect**	−0.241	0.038	−0.318	−0.172
**URT→WA→WE**	−0.107	0.029	−0.166	−0.052
**URT→NA→WE**	−0.079	0.023	−0.132	−0.040
**URT→WA→NA→WE**	−0.055	0.014	−0.083	−0.030

The indirect effect value of negative affect between unreasonable tasks on work engagement was 0.079, and the 95% CI [−0.132 to −0.040] did not contain 0, indicating a significant mediating effect of negative affect between unreasonable tasks and work engagement. Thus, H3c was supported.

The value of the chain mediating effect of work alienation and negative affect was −0.055, and the 95% CI [−0.083 to −0.030] did not contain 0, indicating a significant chain mediating effect. Thus, H4 was supported.

**FIGURE 2:**
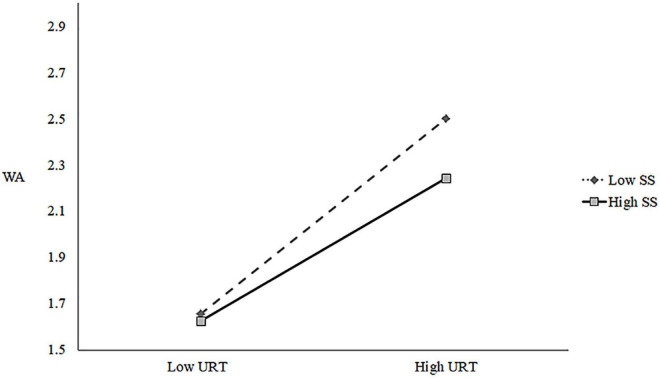
Moderating effect of SS on relationship between URT and WA.

For the moderating effect of supervisor support, we used SPSS 28 to perform a hierarchical regression to test this. In order to cut down multicollinearity, this study centered the two variables of unreasonable tasks and supervisor support before conducting hierarchical regression, and the test results are shown in Table 6. As shown in Model 4 of Table 6, the interaction term of unreasonable tasks and supervisor support had a negative predictive effect on work alienation (β = −0.115, *p* < 0.001). Also, to further visualize the moderating effect, simple slope plots were drawn. As shown in Figure 2, the positive relationship between unreasonable tasks and work alienation diminishes as the level of supervisor support increases. In summary, H5a was supported.

**TABLE 6:** Results of the test for moderating effects.

Variables	WA			
	Model 1	Model 2	Model 3	Model 4
**Sex**	0.020	0.049	0.079	0.070
**Age**	–0.070	0.012	0.006	0.006
**Edu**	0.122*	0.092	0.092	0.076
**JT**	–0.013	–0.006	–0.007	–0.006
**URT**		0.454***	0.422***	0.423***
**SS**			−0.156***	−0.143***
**URT × SS**				−0.115***
***R* ^2^**	0.016	0.258	0.294	0.319
**Δ *R*^2^**	0.016	0.242	0.036	0.026
**F**	1.703	29.227***	29.109***	28.098***

*p < 0.05 and ***p < 0.001.

In this study, model 83 in the PROCESS macro was chosen to test the moderated chain mediating effect. The results showed that the value of the chain mediated effect was −0.068, 95% CI [−0.105 to −0.039] excluding 0 when the level of supervisor support was low; the value of the chain mediated effect was 0.034, 95% CI [−0.059 to −0.015] excluding 0 when the level of supervisor support was high; the difference between high and low groups was 0.034, 95% CI [0.013, 0.063] does not contain 0. In summary, the chain mediating effect diminishes when the level of supervisor support is high and increases when the level of supervisor support is low. In summary, H5b was supported.

## Discussion

The study investigated the association between unreasonable tasks and work engagement. In addition, the mediating role of work alienation and negative affect was examined. Supported by theoretical derivation and data analysis, the research team found that unreasonable tasks have a negative influence on employees’ work engagement. Furthermore, the relationship between unreasonable tasks and work engagement was mediated individually and in a chain by work alienation and negative affect. Finally, we also found a moderating role of supervisor support in the model, specifically that supervisor support negatively moderated the chain mediating role of work alienation and negative affect.

### Theoretical significance

First, the influence mechanism of unreasonable tasks was analyzed in depth from cognitive and affective perspectives to break through the limitations of previous studies. Previous studies on the influence of unreasonable tasks on work engagement have focused on the direct relationship between the two (Schmitt et al., 2015; Kilponen et al., 2021), and some scholars have explored whether there is a mediating effect of self-determined motivation between the two, but the hypothesis was not supported by the data results (van Schie et al., 2014). Therefore, these studies do not fully explain the underlying mechanisms of the impact of unreasonable tasks, which leads to the limited explanatory power of the studies. According to cognitive-affective systems theory, the increase in work alienation reflected employees’ cognitive separation from work, and the generation of negative affect indicated employees’ affective reactions to work. The separate mediating roles of work alienation and negative affect were verified by incorporating cognition and affect into the research model. This research also verified the integrative role of work alienation and negative affect in the process of unreasonable tasks that negatively influence work engagement. The findings of this research can help the academic community understand the mechanism of unreasonable tasks affecting work engagement and widen the application of cognitive-affective systems theory.

Secondly, the present study proposes and validates work alienation as a novel mechanism, which fills the research gap. Existing studies have verified the effects of unreasonable tasks on aspects of mental health, emotional, workplace injuries, and work meaning (Elfering et al., 2018; Koch and Adler, 2018; Pindek et al., 2019; Fila and Eatough, 2020; Mäkikangas et al., 2021), but they lack attention to work alienation. Through empirical analysis, this research confirmed that work alienation is one of the effects of unreasonable tasks and disclosed the “black box” of unreasonable tasks on work engagement. In addition, much of the existing literature includes work alienation as an independent or dependent variable in the research model (Zoghbi-Manrique-de-Lara and Viera-Armas, 2019; Guo et al., 2021; Vanderstukken and Caniels, 2021; Ali et al., 2022). This study used work alienation as a mediating variable and confirmed that this negative perception of work by employees is the transmission mechanism by which work stress influences employees’ behaviors and attitudes. This finding supports the adaptability of work alienation in the Chinese context and further deepens the understanding of the antecedents and consequences of work alienation in the academic community.

Third, the current study verified the moderating effect of supervisor support as a boundary condition on the role of chain mediation and enriched the application of the job demands-resources model. From the beginning of the literature related to unreasonable tasks to the present, the academic community has searched for conditions on how to reduce the adverse effects of unreasonable tasks, which include flexible role orientation, relational transparency, hostile attribution bias, and job crafting (van Schie et al., 2014; Muntz et al., 2019; Pindek et al., 2019; Mäkikangas et al., 2021). These studies have contributed to relevant theories but have not considered intervention conditions from a supervisor’s perspective. Employees consume resources when confronted with unreasonable tasks, and supervisor support can give employees adequate resources that can reduce employees’ negative perceptions of unreasonable tasks. In addition, there is also a study exploring the interaction between supervisor support and unreasonable tasks (Fila and Eatough, 2020). However, the interaction of cognitive variables and chain mediators was lacking and needed to be explored. The present study found that supervisor support as an important source of resources effectively mitigated the negative effects of unreasonable tasks on employees’ work alienation, negative affect, and work engagement, which coincides with the research outlook of Semmer and others on the use of resources as boundary conditions (Semmer et al., 2019) and is also a valid extension of the job demands-resources model.

Lastly, while most studies have focused on the illegitimate task as a whole (Semmer et al., 2010; Meier and Semmer, 2018; Wan et al., 2021; Zhao et al., 2021; Chen et al., 2022), it is valuable to examine the unreasonable task as a separate construct. This research has identified specific characteristics of the unreasonable task that activate the “cognitive-affect-behavior” chain reaction at the theoretical and data levels, which helps to reinforce the understanding of the illegitimate task as a whole and its sub-dimensions, and provides some reference for future research to clarify the nature and specific characteristics of illegitimate tasks’ sub-dimensions.

### Practical significance

As the results of this study show that unreasonable tasks are the cause of reduced employee engagement, it is vital to avoid them as much as possible. Unreasonable tasks come from the organization or the supervisor, so the organization or the supervisor must take each task assigned to the employee seriously and make sure that the tasks are as reasonable as possible for the employee. In this case, the organization should implement some interventions to evade unreasonable tasks at the source, such as redesigning jobs to make tasks more reasonable, modifying job descriptions to widen the scope of employee responsibilities, encouraging employees to participate in the decision-making process of task assignment, and actively listening to employee suggestions.

It is worth mentioning that the data for this study are from a single economy, China. China has grown rapidly over the past two decades, and competition among organizations has become more intense. With this comes an increase in the workload of employees, and the share of unreasonable tasks in it has increased, which makes unreasonable tasks unavoidable in Chinese organizations (Zeng et al., 2021). This study has demonstrated that unreasonable tasks can negatively affect employees, so we hope that the results can provide some help for managers of organizations in China or even abroad. In situations where unreasonable tasks are unavoidable, the question of how to reduce negative effects is a matter of consideration. Research has identified the role of work alienation, negative affect, and supervisor support in the impact of unreasonable tasks, which demonstrates the importance of cognition, affect, and resources on work attitudes and behaviors. First, effective communication can reduce employees’ negative perceptions of unreasonable tasks (Minei et al., 2018). Supervisors enhance communication with employees to give justification for tasks, which can make employees feel that their work is meaningful to the organization and themselves. Supervisors can provide justification to explain why the task needs to be addressed and acknowledge that the task is unreasonable for the employee, thus minimizing the creation of work alienation. Second, organizations and supervisors should pay attention to changes in employees’ affect, actively appease their emergence of negative affect, and often organize leisure activities to allow employees to relax to better engage in their work. At the same time, the organization can consider strengthening the psychological training of employees to improve their affect management skills and psychological quality and reduce the possibility of negative affect arising from unreasonable tasks. Finally, supervisors need to provide resources to support employees to complete their tasks, actively communicate, find out the various special situations of employees, comfort and encourage employees to face work difficulties, and care about employees’ physical and mental health and work-family balance, so that employees have no worries and can devote themselves more to their work.

### Limitations and future research prospects

Any study has both defects and merits, and the limitations of this study are: First, as with any survey, all of the data in this survey came from self-reports. The unreasonableness of tasks is exceptionally subjective, and even core tasks may be considered unreasonable by employees in specific contexts, so unreasonable tasks are hard to determine objectively. One study found a significant difference between supervisors and employees who assessed a lower level of illegitimate task convergence (Meier and Semmer, 2018), implying that it is not a good choice for supervisors to assess employees’ unreasonable tasks. As a result, future research using experimental methods may better measure unreasonable tasks. Secondly, research has shown that experiences with illegitimate tasks vary by culture (Semmer et al., 2015; Ahmed et al., 2018). Eastern countries have high power distance and collectivism, and the values of obedience and self-sacrifice may contribute to employees’ greater willingness to accept unreasonable tasks. Accordingly, our employees report fewer unreasonable tasks than employees in other countries. The findings of this study cannot be generalized to other countries, and future studies may consider multicultural contexts.

## Conclusion

Based on the cognitive-affective systems theory and job demands-resources model, this study constructs a chain mediating model of unreasonable tasks affecting work engagement and explores the moderating role of supervisor support. The study confirms that unreasonable tasks have a negative effect on work engagement. Specifically, unreasonable tasks act as a negative situational factor that activates employees’ cognitive units (work alienation) and affective units (negative affect), thereby exerting a disincentive effect on work engagement. In addition, supervisor support as a boundary condition is effective in mitigating a range of harmful effects from unreasonable tasks.

## Data availability statement

The original contributions presented in this study are included in the article/Supplementary material, further inquiries can be directed to the corresponding author.

## Ethics statement

Ethical review and approval was not required for the study on human participants in accordance with the local legislation and institutional requirements. The patients/participants provided their written informed consent to participate in this study. Written informed consent was obtained from the individual(s) for the publication of any potentially identifiable images or data included in this article.

## Author contributions

HC, ZL, and JZ worked together to discuss the research process, revise the manuscript, and organized the materials. WW and RZ processed the data and wrote the data analysis section. All authors contributed to the article and approved the submitted version.
